# Influence of the Encapsulating Agent on the Bioaccessibility of Phenolic Compounds from Microencapsulated Propolis Extract during *In Vitro* Gastrointestinal Digestion

**DOI:** 10.3390/foods13030425

**Published:** 2024-01-28

**Authors:** Inés Cea-Pavez, David Manteca-Bautista, Alejandro Morillo-Gomar, Rosa Quirantes-Piné, José L. Quiles

**Affiliations:** 1 Research and Development Functional Food Centre (CIDAF), Health Science Technological Park, Avenida del Conocimiento 37, 18016 Granada, Spain; ines.cea@cidaf.es (I.C.-P.); dmanteca@cidaf.es (D.M.-B.); alemogo@correo.ugr.es (A.M.-G.); jlquiles@ugr.es (J.L.Q.); 2 Faculty of Pharmacy, University of Granada, Cartuja Campus, 18071 Granada, Spain; 3 Department of Analytical Chemistry, Faculty of Sciences, University of Granada, Avda Fuentenueva s/n, 18071 Granada, Spain; 4 Department of Physiology, Institute of Nutrition and Food Technology “José Mataix Verdú”, Biomedical Research Centre, University of Granada, 18016 Armilla, Spain; 5 Research Group on Foods, Nutritional Biochemistry and Health, Departamento de Ciencia de los Alimentos y Tecnología Química, Facultad de Ciencias, Universidad Europea del Atlántico, Isabel Torres 21, 39011 Santander, Spain

**Keywords:** propolis, encapsulation, spray-drying, bioaccessibility, *in vitro* gastrointestinal digestion, HPLC-ESI-QTOF-MS/MS

## Abstract

The aim of this work is to develop different encapsulated propolis ingredients by spray-drying and to evaluate their bioaccessibility using simulated *in vitro* digestion. To achieve these goals, first, microparticles of a propolis extract with inulin as the coating polymer were prepared under the optimal conditions previously determined. Then, a fraction of inulin was replaced with other encapsulating agents, namely sodium alginate, pectin, and chitosan, to obtain different ingredients with controlled release properties in the gastrointestinal tract. The analysis of the phenolic profile in the propolis extract and microparticles showed 58 compounds tentatively identified, belonging mainly to phenolic acid derivatives and flavonoids. Then, the behavior of the free extract and the formulated microparticles under gastrointestinal conditions was studied through an *in vitro* gastrointestinal digestion process using the INFOGEST protocol. Digestion of the free extract resulted in the degradation of most compounds, which was minimized in the encapsulated formulations. Thus, all developed microparticles could be promising strategies for improving the stability of this bioactive extract under gastrointestinal conditions, thereby enhancing its beneficial effect.

## 1. Introduction

Currently, consumers adopting a healthier lifestyle are looking for natural, value-added, and healthful functional foods and ingredients. Thus, functional food ingredients and natural food preservatives are of great interest in the food industry [1,2]. Propolis is a honeybee product made from wax, salivary secretions, and resinous material collected from the flowers and leaf buds of plants [3,4,5]. It is composed of resins (50–55%), waxes (25–35%), volatile oils (10%), pollen (5%), and other organic and mineral substances (5%) [6]. Within the fraction of other organic substances, there can be found a wide range of biologically active compounds such as polyphenols, the most important flavonoids, phenolic acids, lignans, and stilbenes, in addition to terpenoids, mainly mono- and sesquiterpenes [7,8,9]. However, the content of these bioactive compounds in propolis depends on the vegetable source from which the bees collect resins; therefore, the geographical location determines the composition of propolis, as it is related to the native vegetation [10]. These compounds are considered responsible for the wide range of beneficial biological activities described for propolis [11], such as anti-inflammatory, antioxidant, hepatoprotective, antiulcerogenic, cytotoxic, immunostimulant, antifungal, and antibacterial properties [12,13,14]. 

Many procedures for extracting bioactive compounds from propolis can be found in the scientific literature based on different techniques, both conventional and non-conventional. All these different procedures affect the extraction yield or extraction time, but the composition of the obtained extract generally does not significantly change [15,16]. The traditional method to extract bioactive compounds from propolis involves macerating the propolis in a 70% aqueous ethanol solution. This method extracts the resin fraction, which contains the main bioactive compounds [17], including the most important flavonoids such as pinobanksin, pinocembrin, chrysin, galangin, kaempferide, or kaempferol, among others, as well as phenolic acids such as caffeic, cinnamic, p-coumaric, or ferulic acids artepellin C and drupanin, in addition to esters of these acids such as bacarin, benzyl caffeate, phenethyl caffeate, and benzyl hydroxybenzoate [18]. 

However, the main limitations to the implementation of these functional extracts as food ingredients are their relatively low stability and bioavailability. Therefore, it is of primary importance to assess if the intact bioactive compounds reach their absorption sites, mainly the colon in the case of phenolic compounds, since they could suffer degradation under the unfavorable conditions that occur during the digestion process, such as high temperature, low pH and enzymatic activities.

To prevent this degradation, processing techniques such as spray-drying encapsulation can be used. This is one of the most used technologies on an industrial scale because it is rapid, low-cost, and reproducible [19,20]. In this technique, a liquid (solution, dispersion, or emulsion) is atomized in a drying medium that is usually hot air, instantly obtaining a powder product due to the rapid evaporation of the solvent. The particles are collected after they fall to the bottom of the cyclone, with sizes between 10 and 100 μm [19,20,21]. One of the advantages of this technique, in addition to its simplicity, is its usefulness for encapsulating thermosensitive materials because of the short period of exposure to high temperatures (between 5 and 30 s) [22]. As a result of this process, bioactive compounds are covered with a polymer matrix that protects them from degradation caused by external agents. Depending on the coating material chosen, the content of the microparticles can be released in different parts of the gastrointestinal tract [19]. 

Recently, the encapsulation of propolis extracts by spray-drying has been studied, using mainly polymers such as maltodextrin and proteins as encapsulating agents [20,21,22]. However, there are no studies related to propolis encapsulation using coating materials that enable a controlled release of the active ingredients in a specific area of the gastrointestinal tract. A low number of polymers can be used to protect bioactive components from harmful conditions in the stomach and upper part of the small intestine, allowing the release of bioactive components through the action of the colonic microbiota [23]. The most used polymers with controlled release properties are chitosan, alginate, pectin, dextrans, starch, and inulin, all of them insoluble polysaccharides which can act as substrates for the inhabiting bacterial microbiota in the intestine [21,23]. 

In relation to the polymers used, inulin (IN) and pectin (P) are polysaccharides advantageous for targeted delivery because they move through the stomach and small intestine intact, whereas their degradation largely depends on enzymes derived from the microbiota host [21,23]. However, sodium alginate (SA) and chitosan (CH) are pH-dependent release polymers. The alginate shrinks at low pH (gastric conditions), so the encapsulated compounds are not released. In the gastric fluid, the hydrated sodium alginate becomes an insoluble viscous layer called alginic acid. However, once it passes to a higher pH (intestinal conditions), this alginic acid becomes a soluble viscous layer [24]. This pH-dependent behavior can be exploited to modify the release profiles by using it as an encapsulating agent because it can be applied to facilitate the release of bioactive components in the ileum or colon [21]. Chitosan easily dissolves at low pH, but it is often applied in combination with another polymer that withstands low pH in the stomach, such as alginate. Upon arrival in the gut, the rich colonic microbiota degrades this polymer [21].

In this context, the objective of this work is to investigate the effect of different coating materials on the encapsulation of a propolis extract and their behavior during simulated *in vitro* digestion in the stomach and small intestine. 

## 2. Materials and Methods

### 2.1. Chemicals

All reagents and solvents were of analytical or MS grade. For extraction, n-hexane and absolute ethanol (EtOH) were procured from Fisher Scientific (Loughborough, Leicestershire, UK). LC–MS grade acetonitrile from Fisher Scientific, formic acid from Sigma-Aldrich (Saint Louis, MO, USA), and phenolic compounds standards from Sigma-Aldrich or Extrasynthese (Lyon, France) were used for HPLC-MS analyses. Gallic acid and Folin–Ciocalteu reagent were also from Sigma-Aldrich. For antioxidant activity assays, sodium acetate anhydrous and glacial acetic acid were purchased from Fisher Scientific, whereas ferric chloride anhydrous, Trolox^®,^ and 2,4,6-Tris(2-pyridyl)-s-triazine were from Sigma-Aldrich.

Regarding polymers used for the synthesis of the microparticles, inulin, sodium alginate, and chitosan were purchased from Guinama S.L.U. (La Pobla de Vallbona, Valencia, Spain), and pectin was purchased from Fragon Ibérica (Terrasa, Barcelona, Spain). Enzymes for *in vitro* digestion (pepsin 3412 U/mg protein and pancreatin 4 × USP) and bovine bile salts were obtained from Sigma-Aldrich. Hydrochloric acid, sodium hydroxide, potassium chloride, sodium hydrogen carbonate, potassium dihydrogen phosphate, ammonium carbonate, and sodium chloride were obtained from Fisher Chemicals (Waltham, MA, USA) for preparation of the simulated digestive fluids. Ultrapure water was obtained with a Milli-Q system (Millipore, Bedford, MA, USA).

### 2.2. Sample

The propolis sample was provided by Apícola Valle del Maitena-Apicultura El Cañuelo (Güéjar Sierra, Granada, Spain). It was necessary to dewax the propolis sample before its encapsulation, and then, phenolic compounds were extracted by maceration with ethanol:water (70:30, v/v) as described by Cea-Pávez et al. [25]. The propolis extract (PE) obtained was stored at −20 °C in dark conditions until its microencapsulation.

### 2.3. Formulation of Controlled Release Ingredients by Spray-Drying PE Encapsulation

Several ingredients with properties of controlled release in the gastrointestinal tract were developed by using as encapsulating agents only inulin (PE-IN) or mixtures with other polymers by replacing 10% of inulin with other encapsulating agents such as sodium alginate (PE-IN/SA), pectin (PE-IN/P), and chitosan (PE-IN/CH). The microencapsulation conditions were optimized in a previous study [25]. Briefly, the spray-dryer 4M8-TriX instrument (ProCept, Zalzate, Belgium) was operated at 112.65 °C inlet temperature, PE/encapsulating agent ratio of 1:4.315, air flow 0.4 m^3^/min, feed rate at 2 mL/min (10%), and nozzle air at 20 L/min.

### 2.4. Characterization of the PE Microparticles

PE microparticles were characterized according to the encapsulation efficiency (EE) and the Recovery 1 and Recovery 2 values of different phenolic compounds calculated using Equations (1), (2), and (3), respectively. The total and superficial contents of the microparticles were extracted as described in detail elsewhere [25].

(1) $$EE\;\left(\%\right)=\frac{Experimental\;total\;content\;\left(\mathrm{mg}\right)-superficial\;content\;(\mathrm{mg})}{Experimental\;total\;content\;(\mathrm{mg})\;}\times 100$$

(2) $$Recovery\;1\left(\%\right)=\frac{Experimental\;total\;content\;in\;the\;powder\;(\mathrm{mg})}{Experimental\;total\;content\;in\;the\;feed\;solution\;(\mathrm{mg})}\times 100$$

(3) $$Recovery\;2\left(\%\right)=\frac{Experimental\;total\;content\;in\;the\;powder}{Theorical\;total\;content\;in\;the\;extract\;in\;the\;feed\;solution}\times 100$$

Additionally, the total content of phenolic compounds (TPC) was measured with the Folin–Ciocalteu method [26], and the total flavonoid content (TFC) [27] and antioxidant capacity were measured using FRAP [28] and ORAC assays [29].

### 2.5. In Vitro Gastrointestinal Digestion

Static *in vitro* gastrointestinal digestion was performed using the INFOGEST 2.0 method [30]. To replicate oral digestion, 5 g of PE, PE-IN, PE-IN/SA, PE-IN/P, and PE-IN/CH were resuspended in 5 mL (1:1, w/v) of Simulated Salivary Fluid (SSF) in a 50-mL centrifuge tube. This mixture was stirred for 5 min, protecting it from light. 

For the gastric phase simulation, the bolus was mixed with 7.5 mL of Simulated Gastric Fluid (SGF), 2000 U/mL of pepsin, and 5 µL of CaCl_2_ 0.3 M. The pH was adjusted to 3.0 by adding the necessary volume of 1 M HCl. The final volume for this step was adjusted to 18 mL by adding Milli-Q H_2_O. 

The gastric phase was performed for 120 min at 37 °C under constant agitation at 150 rpm using an incubator (MaxQTM 6000 SHKE6000-8CE, Thermo Scientific, Waltham, MA, USA). 

The intestinal phase was prepared by adding 9.8 mL of Simulated Intestinal Fluid (SIF), 100 U/mL of pancreatin, 2.5 mL of bile, and 40 µL of CaCl_2_ 0.3 M to the existent simulated chyme. Then, the pH was fixed to 7.0, adding the required volumes of 1 M NaOH, and Milli-Q H_2_O was added to achieve a final volume of 40 mL. This was homogenized. The intestinal phase was carried out for 30, 60, and 120 min at 37 °C under conditions of constant agitation at 150 rpm using an incubator (MaxQTM 6000 SHKE6000-8CE, Thermo Scientific, Waltham, MA, USA). 

All static digestion experiments were performed in duplicate.

### 2.6. HPLC-MS Analyses 

Phenolic compounds analyses in PE, microparticles, and digested samples were carried out using High-Performance Liquid Chromatography Coupled to Electrospray Quadrupole-Time-of-Flight Mass Spectrometry (HPLC-ESI-QTOF-MS/MS) (HPLC 1260 coupled to 6540 Ultra High Definition Accurate-Mass Q-TOF, Agilent Technologies, Palo Alto, CA, USA) with the method described elsewhere [25]. Briefly, the separation was performed with a Agilent Zorbax Eclipse Plus C18 column (150 mm × 4.6 mm, 1.8 µm) using as mobile phases water with 0.1% formic acid and acetonitrile in gradient elution mode. Detection was performed in negative ion mode within a mass range of 50–1700 m/z.

### 2.7. Gastrointestinal Digestion Recovery and Bioaccessibility

Digestion recovery and bioaccessibility of different phenolic compounds, defined as the amount of compound that is released from the matrix in each phase of the digestion and after complete digestion, respectively [31], were calculated using Equations (4) and (5). Individual contents in every compound during and before digestion were quantified with HPLC-MS according to the methodology previously described.

(4) $$Recovery\;\left(\%\right)=\frac{Experimental\;total\;content\;during\;digestion\;of\;the\;product\;(\mathrm{mg})}{Experimental\;total\;content\;in\;product\;before\;digestion\;(\mathrm{mg})}\times 100$$

(5) $$Bioaccessibility\;\left(\%\right)=\frac{Experimental\;total\;content\;after\;complete\;digestion\;product\;(\mathrm{mg})}{Experimental\;total\;content\;in\;product\;before\;digestion\;(\mathrm{mg})}\times 100$$

### 2.8. Statistical Analysis

The results are expressed as means ± standard deviations. One-way analysis of variance (ANOVA) was performed for EE, Recovery 1, Recovery 2, TPC, TFC, FRAP, ORAC, and gastric digestion recovery. Two-way analysis of variance (ANOVA) and Duncan multiple range analysis were carried out on intestinal digestion recovery. All statistical analyses were performed using Statgraphics Centurion XV (StatPoint Inc., Warrenton, VA, USA, 2011). Differences were considered significant when *p* < 0.05.

## 3. Results and Discussion

### 3.1. PE Preparation and Characterization Using HPLC-MS

**Figure 1:**
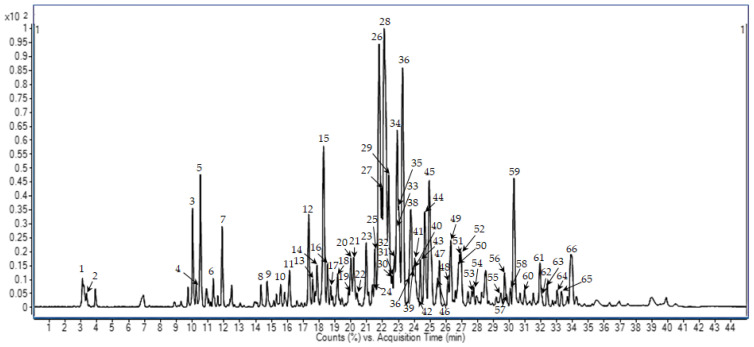
Base Peak Chromatogram (BPC) of PE at a concentration of 5 mg·mL^−1^. Detected peaks were numbered according to their elution order, and these numbers correspond to those compounds identified in Table 1.

The propolis sample was dewaxed and extracted as described by Cea-Pavez et al. [25]. The resulting PE was reconstituted at a concentration of 5 mg·mL^−1^ in aqueous ethanol 70% and analyzed using HPLC-ESI-QTOF-MS/MS, obtaining the chromatogram shown in Figure 1. The main compounds were automatically detected with a molecular feature extraction algorithm applying a volume threshold of 0.3% with respect to the main peak. As a result, 66 compounds were detected and tentatively identified whenever possible by interpretation of their MS and MS/MS spectra and comparison with databases and the previous literature. Among the detected compounds, 58 could be identified in the propolis extract. All detected compounds with their MS data and putative identification are detailed in Table 1.

The majority of identified compounds were phenolic acids and flavonoids, although a few terpenoids, fatty acids, and carbohydrate derivatives were also in the extract. Because this study focused on phenolic compounds, the main compounds belonging to this family were also quantified. To perform this quantitative analysis, phenolic compounds with a peak volume above 0.5% were selected for quantification. Because of the great diversity of phenolic compounds, no commercial standards were available for all of them. Therefore, a common approximation was applied for quantification using surrogate standard compounds with a similar chemical structure. In this way, standard calibration curves of 7 points of caffeic acid, p-coumaric acid, naringenin, apigenin, kaempferide, quercetin, chrysin, sakuranetin, pinocembrin, and luteolin were prepared and analyzed in triplicate. The chromatographic area of the detected peak for each compound was later interpolated on the corresponding calibration curve of the selected standard based on structural similarity, obtaining the calculated concentration. The content in mg/g of dry extract was calculated for each triplicate, and the resulting data expressed as mean value ± standard deviation are included in Table 1.

The total concentration of all quantified compounds represents more than 950 mg/g of dry extract, which confirms that the most representative compounds of the extract have been properly quantified. These quantitative results show that the major compounds correspond to phenolic acid derivatives, mainly caffeic and coumaric acid derivatives, as well as flavonoids, such as pinobanksin, chrysin, apigenin, pinocembrin, and their derivatives. The two major compounds were prenyl caffeate isomers (431 ± 4 and 191 ± 6 mg/g), representing approximately 60% of the composition of the extract, followed by both isomers of dupanin, pinobanksin acetate and β-phenylethyl caffeate, which showed concentrations of approximately 50–30 mg/g.

The composition of the obtained and analyzed PE did not considerably differ from other propolis extracts previously described in the scientific literature [32,33,34,35,36,37,38,39,40,41,42,43,44,45]. Some authors found that phenylethyl caffeate, chrysin, and pinocembrin were some of the major compounds in propolis from different zones (Italy, China, Argentina, Ukraine, and Macedonia) [41], which is in agreement with our results.

### 3.2. Encapsulation of PE by Spray-Drying and Characterization of Microparticles

PE was encapsulated by spray-drying using inulin and mixtures of inulin with sodium alginate, pectin, or chitosan in a 9:1 proportion, as previously described in Section 2.3. In this way, PE-IN, PE-IN/SA, PE-IN/P, and PE-IN/CH microparticles were prepared, and the effect of the different polymers on the encapsulation efficiency and recovery of individual phenolic compounds was assessed. These results are presented in Table 2 and Table 3, respectively.

As shown in Table 2, EE% varied between 30.3 and 86.7% for PE-IN, 40.6 and 90.6% for PE-IN/SA, 24.3 and 75.5% for PE-IN/P, and 16.3 and 85.2% for PE-IN/CH. Comparing PE-IN/SA with PE-IN, it is observed that EE is similar in most phenolic compounds with a few exceptions, such as pinobanksin and its derivatives, as well as both isomers of prenyl caffeate, where there was an increase in EE. Regarding PE-IN/P, EE was the same or decreased with respect to PE-IN microparticles. Finally, the PE-IN/CH formulation presented a different behavior because the effect of replacing a fraction of inulin with chitosan over EE depended on the compound. In this way, some compounds showed similar EE, whereas this value decreased in other compounds, such as caffeic acid, apigenin, or kaempferol, and increased in some flavonoid compounds, such as rhamnetin and dimethylquercetin. Therefore, the EEs were significantly different between microparticles, showing that the effect of the different encapsulating agents used due to EE may be related to the interaction between the bioactive compounds and the encapsulating polymers [46]. This effect has been previously observed in the microencapsulation of propolis extracts using other coating materials [47,48].

Recovery of the different phenolic compounds is summarized in Table 3. Recovery 1 (calculated from the phenolic compounds present in the feed solution) varied between 66.7 and 128.0% for PE-IN, 21.6 and 41.5% for PE-IN/SA, 35.0 and 63.6% for PE-IN/P, and 35.5 and 139.6% for PE-IN/CH. Significant differences were observed between PE-IN and microparticles with polymer mixtures (PE-IN/SA, PE-IN/P, and PE-IN/CH). When comparing PE-IN with PE-IN/SA and PE-IN/P, it was observed that the latter decreased the recovery of all phenolic compounds. On the other hand, when compared with PE-IN/CH, only a few phenolic compounds suffered this reduction; others remained similar, whereas other compounds presented a rise in recovery, such as prenyl caffeate and drupanin. These results mean that the mixture of the studied polymers reduces the protection of the different bioactive compounds under drying conditions.

On the other hand, the results of Recovery 2 (calculated from the phenolic compounds present in the PE used for the preparation of the different feed solutions) showed values that varied between 21.2% and 129.8% for PE-IN, 36.2% and 76.1% for PE-IN/SA, 34.6% and 100.7% for PE-IN/P, and 63.4 and 104.3% for PE-IN/CH. Again, significant differences were observed between PE-IN and microparticles with polymer mixtures. The addition of SA and CH resulted in higher recovery values of all phenolic compounds compared with microparticles encapsulated only with IN, whereas the addition of P did not affect recovery. These findings could be explained by an increase in the dispersibility of PE in the feed solution because the three-dimensional network that forms the polymer mixture (IN/SA and IN/CH) prevents PE from precipitating [49,50].

Furthermore, the microparticles were characterized in terms of total phenolic and flavonoid contents (TPC and TFC) as well as *in vitro* antioxidant activity determined by ferric reducing antioxidant power (FRAP) and oxygen radical absorbance capacity (ORAC) assays. The results are presented in Table 4, and they confirmed that PE-IN/SA, PE-IN/P, and PE-IN/CH microparticles had a significantly higher content of bioactive compounds and greater antioxidant capacity than PE-IN. These results are in agreement with the greater Recovery 2 found in the microparticles with polymer mixtures, which would also explain the greater antioxidant capacity. In addition, the correlations between TPC/FRAP, TPC/ORAC, TFC/FRAP, and TFC/ORAC were studied, obtaining a positive correlation in TPC/ORAC and TFC/ORAC with an r^2^ of 0.989 and 0.6876, respectively.

### 3.3. In Vitro Gastrointestinal Digestion of Microparticles

The propolis extract as well as the different designed microparticles, were subjected to *in vitro* simulation of the digestive process in the mouth and stomach (gastric digestion) and small intestine (intestinal digestion). The recovery of each individual compound grouped by family after 2 h of gastric digestion is presented in Table 5, and recovery along intestinal digestion (at 30, 60, and 120 min of intestinal phase), including bioaccessibility at the end of the process, is presented in Table 6 and Table 7. In the next subsections, the behavior of the main families of compounds in each assayed formulation will be discussed.

#### 3.3.1. Caffeic Acid Derivatives

In the case of the free extract, the recovery of the major and more complex derivatives, such as the two isomers of prenyl caffeate, β-phenylethyl caffeate and isopentyl caffeate, was very limited and decreased throughout the digestive process. This indicates a degradation of these complex compounds under digestion conditions to give rise to simpler compounds such as caffeoylglycerol and caffeic acid itself. This degradation would explain the recovery values greater than 100% for both simple compounds, which decreased as digestion progressed, showing their maximum concentration in the gastric phase and subsequently decreasing throughout the intestinal phase. These results demonstrated the need to protect these types of compounds so that they can reach the colon unaltered. The limited stability of different phenolic compounds, including phenolic acids such as caffeic or p-coumaric acids, under simulated gastrointestinal conditions, has been previously reported by Li et al. [51], although the bioaccessibility values described in this previous study for different phenolic acids were slightly higher than those obtained for caffeic acid derivatives in PE. These lower values may be attributed to the functionalization of these derivatives, which could negatively affect their stability under gastrointestinal conditions. These results are also in agreement with those previously reported by Yen et al. [52], who observed a reduction in the release of total phenolics and flavonoids in the gastric and intestinal phases of a freeze-dried brown propolis extract, although these authors attributed this decrease to possible enzymatic interactions because data on individual compounds were not available.

On the other hand, if the recovery data of the different microparticles are compared, the first difference that should be highlighted is that both caffeoylglycerol and caffeic acid presented lower recoveries in all cases. Indeed, they did not exceed 100% except at the end of the intestinal phase in the PE-IN/SA particles, where caffeic acid had a value slightly higher than 100%. These results indicate a drastic decrease in the degradation of the more complex caffeic acid derivatives compared with the free extract. Although this tendency is common to all microparticles, the individual behavior of each compound differs greatly between some formulations and others. In the case of PE-IN particles, the recoveries after gastric digestion of these compounds were quite low, with values between 7% and 20% for complex derivatives and around 50% for caffeoylglycerol and caffeic acid, and in most cases, lower than the values measured in the free extract. However, at the end of their intestinal digestion, their bioaccessibilities significantly increased, except in the case of caffeoylglycerol, which remained constant. These results suggest a poor release of microparticles under stomach conditions and that the low gastric recoveries were likely due to the superficial phenolic compound content in the microparticles. Regarding intestinal recoveries, although inulin is relatively stable to hydrolysis at neutral pH, a partial release of the content of inulin-based microparticles under intestinal conditions has also been previously reported for encapsulated olive leaf extract, and this release has been attributed to Fickian diffusion [53].

The behavior of the PE-IN/SA microparticles in terms of trend is very similar, although the recovery values in the gastric phase were significantly lower. Therefore, a more pronounced increase was observed in the intestinal phase, although in some cases, the value obtained was lower than the bioaccessibility of the compounds in the PE-IN particles. This behavior was expected because sodium alginate shrinks at low pH, making the microparticles more stable in the gastric environment, but it swells under neutral–alkaline pH (intestinal conditions), releasing the encapsulated compounds [54]. However, the combination of this polymer with inulin confers a higher stability to the microparticles, showing a lower release of the phenolic compounds in the intestinal phase than those previously reported for particles developed exclusively with SA to encapsulate oleuropein [55]. This type of interaction between SA and other polysaccharide-based polymers, which reinforce the stability of the microparticles under intestinal conditions, has been previously described for the combination of SA and guar gum [54].

In the case of PE-IN/P particles, gastric recovery was similar to PE-IN, although slightly higher, with values around 18–30% for complex derivatives and around 60% for caffeoylglycerol and caffeic acid. However, along the intestinal phase, the bioaccessibilities of the complex derivatives slightly decreased, whereas a rise was observed for the simplest compounds. This could be explained by a partial release of the microparticle content under gastric conditions and subsequent degradation of the complex derivatives under intestinal conditions without increasing the release of the microparticle content.

PE-IN/CH particles showed very different behavior. For this formulation, low recoveries were measured in the gastric phase, between 1% and 15% in all caffeic acid derivatives. These low values were maintained in the intestinal phase, where recoveries below 5% were obtained for the complex derivatives and between 12% and 14% for caffeoylglycerol and caffeic acid. These results point to practically no release of particles in both gastric and intestinal phases, with the measured concentrations being due to the superficial content of the microparticles. As occurred in the free extract, the superficial complex compounds suffered degradation during the digestive process, decreasing their recovery values in the intestinal compared with the gastric phase, whereas caffeic acid slightly increased due to the degradation of its derivatives. The effectiveness of chitosan as a coating material to prevent the release of encapsulated bioactive compounds has been previously reported for ellagic acid [56] and caffeine [57].

#### 3.3.2. Coumaric Acid Derivatives

The recovery data for coumaric acid derivatives replicated the behavior of caffeic acid derivatives, although the recovery values were generally lower than those of the previously discussed family. These results disagree with previous studies that showed an increase in the bioaccessibility of hydroxyphenolic acids with a decrease in the number of hydroxyl substitutions [51], likely due to differences in the stability according to the substituents in the phenolic acid core. In the free extract, the recoveries of the complex derivatives of coumaric acid were limited, with values lower than 10% in the gastric phase and a slight rise in the intestinal phase of both benzyl p-coumarate and capillartemisin, whereas the two isomers of drupanin reduced by half. However, p-coumaric acid presented recovery values greater than 100% in both phases, indicating a degradation of its complex derivatives. Microparticles formulated with inulin showed the protection of these compounds with values that were generally lower in the gastric phase and higher in the intestinal phase compared with the free extract and with much lower recovery values of p-coumaric acid. The microparticles formulated with SA also presented a similar behavior but with much lower recovery in the gastric phase and similar in the intestinal phase compared with the particles containing only IN, as occurred with the caffeic acid derivatives. In the case of the formulation with pectin, a greater release of the microparticle content was seen in the gastric phase, although always with values lower than 25% for complex derivatives and a value of approximately 50% for p-coumaric acid. During the intestinal phase, these recoveries decreased in the case of the derivatives and significantly increased for p-coumaric acid. Regarding the formulation with chitosan, as described for caffeic acid derivatives, the complex coumaric acid derivatives had very low recoveries in the gastric phase, less than 5%, which decreased in the intestinal phase, while p-coumaric acid increased from 7% to 12%.

#### 3.3.3. Pinobanksin Derivatives

The pinobanksin derivatives quantified in the extract and the formulated microparticles were mostly simple derivatives that did not contain glycosidic groups but were methyl, acetate, or pentanoate derivatives. Only hydroxy methoxyphenylpropenyl pinobanksin had a more complex structure.

Pinobanksin showed very high recovery in the gastric phase for the free extract, although it did not exceed 100%. Therefore, it cannot be claimed that degradation of the derivatives contributed to this high recovery, although all other related compounds showed much lower values after gastric digestion, except pinobanksin-3-O-pentanoate isomer 1, which will be discussed below. Regarding the intestinal phase, both pinobanksin and its methylated derivative decreased their recoveries, indicating partial degradation of these compounds. However, the remaining derivatives, except the previously mentioned pentanoate derivative, showed a rise in bioaccessibility, probably due to greater solubilization of these compounds under intestinal pH conditions and by the formation of micelles by bile salts present in the medium. The compound pinobanksin-3-O-pentanoate isomer 1 had a gastric recovery of 142% that subsequently decreased to 32% in the intestinal phase and which also presented behavior very different from that of its isomer; this could be explained by a possible interconversion process between both isomers favored by acidic pH. This type of isomerization process during gastrointestinal digestion has been previously described for hydroxycinnamic acids, such as ferulic, p-coumaric, and sinapic acids [51], as well as for chlorogenic acid [58]. On the other hand, it should also be noted that this compound had the lowest concentration quantified in the extract; therefore, small differences in concentration, even due to experimental errors, would translate into a large difference in relative magnitudes as recovery.

Assessing the effect of the different microparticle formulations on the recovery of these compounds, the general behavior was similar to that discussed for caffeic acid derivatives. In PE-IN microparticles, gastric recoveries decreased in all compounds, whereas intestinal bioaccessibility slightly increased, although only in the case of pinobanksin did it reach values around 90%, whereas for the remaining compounds, they did not exceed 50%. With respect to PE-IN/SA particles, gastric recovery decreased drastically, whereas intestinal bioaccessibility did not differ greatly from that of the formulation with only inulin, again corroborating the greater protection of sodium alginate against gastric conditions compared with inulin. PE-IN/P formulation presented greater recovery under gastric conditions than the particles that exclusively used IN as the encapsulating agent, with slightly lower intestinal bioaccessibility values except in the case of pinobanksin, in which it remained constant. These results point to a decrease in protection with partial replacement with pectin. PE-IN/CH microparticles, as previously observed, showed very low recovery, both gastric and intestinal, with values lower than 15% in all cases, indicating a very low release of the microparticle content under simulated digestion conditions.

#### 3.3.4. Other Flavonoids

The behavior of the remaining flavonoids, including major compounds such as chrysin, apigenin isomer, and pinocembrin, was very similar to that previously discussed for the other compounds. Within this group, there were also no glycosylated derivatives, dimers, or forms that contained other compounds of this group in their structures, which could lead to an increase in the concentration of some compounds due to degradation processes. In general, the tendency of all of them was to present medium or low recoveries in the gastric phase when they were free, except in the case of rhamnetin/methylquercetin isomer 1, which presented values much higher than 100%. This exceptional behavior could be explained in a manner similar to that of pinobanksin-3-O-pentanoate isomer 1 previously discussed and could be due to interconversions of its isomer. In the free extract, bioaccessibilities in the intestinal phase varied greatly from one compound to another, with, in some cases, an increase observed with respect to the gastric phase, as occurred for chrysin, pinocembrin, and both isomers of apigenin, probably due to an improvement in their solubility under those pH conditions and favored by the micelles formed by bile salts. However, other compounds, such as kaempferol or both isomers of pinostrobin/medicarpin/alpinetin, showed a marked decrease when passing into the intestinal phase, indicating partial degradation of these compounds.

The protective effect of the different formulated microparticles observed on this family of compounds did not vary with respect to that previously discussed. Microparticles formulated exclusively with IN notably decreased gastric recovery and significantly increased intestinal bioaccessibility. The partial replacement of IN with SA provided greater protection during the gastric phase, whereas in the intestinal phase, the differences with respect to PE-IN particles were limited, and differences were only observed in a few compounds. When the partial substitution was made with pectin, there was a marked increase in the gastric recovery of the compounds and a slight reduction in the intestinal bioaccessibility of most flavonoids. When replacing part of the IN with chitosan, as for the rest of the compounds, the decrease in both gastric and intestinal recovery was drastic.

The overall conclusion of the *in vitro* static digestion study of the different formulated microparticles is that all microparticles generally protected all families of compounds from degradation throughout the digestive process, although the microparticles in which IN was partially replaced by chitosan were the ones that exerted a more pronounced protective effect. However, it would be necessary to extend the study to confirm that under colonic conditions, the release of the microparticle content will occur to ensure that bioactive compounds can be absorbed and not excreted. On the other hand, the partial replacement of IN with SA led to microparticles with greater protection during the gastric phase, whereas the partial replacement with P led to a greater release in the gastric phase. The release of the PE-IN/SA microparticle content in the intestinal phase was also lower than that of PE-IN, although the difference was less pronounced than in the gastric phase.

## 4. Conclusions

Propolis extract encapsulation by spray-drying with inulin and mixtures of inulin with other polymers such as sodium alginate, pectin, and chitosan has proven to be a good strategy for designing microparticles with controlled release properties, achieving high encapsulation efficiency and recovery of phenolic compounds. Among the designed microparticles, those developed with inulin/sodium alginate and inulin/pectin showed a higher content of bioactive compounds and greater antioxidant capacity compared with those developed with inulin alone or a combination of inulin/chitosan. Regarding their stability and controlled release under simulated gastrointestinal digestion, all microparticles have been shown to protect all families of compounds from degradation, although formulation with inulin/chitosan exerted a more pronounced protective effect. However, it would be necessary to confirm in future studies the release of microparticles under colonic conditions. The findings of this work confirm that the process carried out (pretreatment, extraction of phenolic compounds from propolis, and its microencapsulation) is a successful methodology for the formulation of natural ingredients that could be incorporated into different food matrices to develop functional foods or directly commercialized as nutraceuticals, which may contribute to the development of bee production and revalorization of this resource.

## Figures and Tables

**Table 1:** Main compounds tentatively identified in PE by HPLC-MS and concentration of those selected for quantification.

Peak	RT (min)	m/z	Molecular Formula	Score	Error (ppm)	Peak Relative Volume (%)	Proposed Compound	Mean ± SD (mg/g)
1	3.09	195.0511	C_6_H_12_O_7_	99.96	−0.22	0.35	Gluconic acid	-
2	3.35	387.1147	C_12_H_22_O_11_	99.94	−0.21	0.31	Disaccharide	-
3	10.04	253.0719	C_12_H_14_O_6_	99.51	−0.78	1.33	Caffeoylglycerol	4.9 ± 0.2
4	10.27	137.0245	C_7_H_6_O_3_	99.89	−0.72	0.31	4-Hydroxybenzoic acid	-
5	10.53	179.0351	C_9_H_8_O_4_	99.84	−0.51	1.92	Caffeic acid	9.8 ± 0.7
6	11.34	281.1034	C_14_H_18_O_6_	98.90	−0.99	0.42	Unknown	-
7	11.90	163.0402	C_9_H_8_O_3_	99.69	−0.99	1.12	p-Coumaric acid	7.2 ± 0.4
8	14.35	415.1039	C_21_H_20_O_9_	98.04	−1.25	0.35	Daidzin	-
9	14.74	207.0665	C_11_H_12_O_4_	99.80	−0.87	0.41	Ethyl caffeate/3,4-Dimethoxycinnamic acid/Methyl ferulate	-
10	15.59	301.0356	C_15_H_10_O_7_	99.72	−0.52	0.38	Quercetin	-
11	16.15	315.0512	C_16_H_12_O_7_	99.48	−0.68	0.59	Rhamnetin/Methyl quercetin isomer 1	1.32 ± 0.04
12	17.36	269.0456	C_15_H_10_O_5_	99.37	−0.38	1.58	Apigenin	3.5 ± 0.2
13	17.61	271.0612	C_15_H_12_O_5_	99.56	−0.11	0.43	Naringenin	-
14	17.89	285.0407	C_15_H_10_O_6_	99.73	−0.86	0.72	Kaempferol	1.85 ± 0.06
15	18.29	271.0612	C_15_H_12_O_5_	99.73	−0.24	3.32	Pinobanksin	8.6 ± 0.4
16	18.55	299.0563	C_16_H_12_O_6_	99.87	−0.55	0.72	Isokaempferide/Diosmetin	1.17 ± 0.04
17	18.74	435.1090	C_24_H_20_O_8_	98.94	−1.07	0.32	Unknown	-
18	19.18	355.1191	C_20_H_20_O_6_	98.73	−1.06	0.57	Pinobanksin-3-O-pentanoate isomer 1	0.58 ± 0.02
19	19.88	301.0721	C_16_H_14_O_6_	99.45	−0.97	0.32	Alnustinol/Hesperetin/Dihydrokaempferide	-
20	20.02	299.0563	C_16_H_12_O_6_	98.36	−1.01	1.00	Kaempferide	-
21	20.19	315.0515	C_16_H_12_O_7_	98.83	−1.54	0.94	Rhamnetin/Methyl quercetin isomer 2	2.8 ± 0.1
22	20.26	233.0822	C_13_H_14_O_4_	99.16	−1.26	0.39	Viscidone	-
23	20.99	329.0671	C_17_H_14_O_7_	99.48	−1.06	1.21	Dimethyl quercetin/Eupalitin	3.3 ± 0.1
24	21.38	251.1655	C_15_H_24_O_3_	99.84	−0.66	0.35	Unknown	-
25	21.53	235.0979	C_13_H_16_O_4_	99.46	−1.12	1.00	Butyl caffeate	4.9 ± 0.2
26	21.80	247.0978	C_14_H_16_O_4_	99.56	−1.08	6.49	Prenyl caffeate isomer 1	191 ± 6
27	21.97	269.0822	C_16_H_14_O_4_	99.29	−1.00	1.91	Pinostrobin/Medicarpin/Alpinetin isomer 1	7.0 ± 03
28	22.14	247.0980	C_14_H_16_O_4_	99.38	−1.48	8.97	Prenyl caffeate isomer 2	431 ± 4
29	22.40	253.0509	C_15_H_10_O_4_	99.21	−0.91	2.85	Chrysin	17 ± 1
30	22.53	285.0771	C_16_H_14_O_5_	99.28	−1.00	0.50	Sakuranetin	4.4 ± 0.2
31	22.63	313.0720	C_17_H_14_O_6_	99.73	−0.77	0.50	Pinobanksin acetate/Dimethyl kaempferol isomer 1	1.43 ± 0.06
32	22.77	283.0616	C_16_H_12_O_5_	85.49	−1.27	0.98	Acacetin/Calycosin/Thevetiaflavone/Biochanin A/Methylgalangin/Pterosonin E/6-Methoxybaicalein isomer 1	6.4 ± 0.3
33	22.90	255.0667	C_15_H_12_O_4_	99.07	−1.37	1.67	Pinocembrin	11.3 ± 0.7
34	22.94	283.0977	C_17_H_16_O_4_	99.47	−0.50	3.32	β-Phenylethyl caffeate	33 ± 2
35	23.04	269.0458	C_15_H_10_O_5_	99.24	−0.91	2.07	Apigenin isomer	15.4 ± 0.8
36	23.28	313.0722	C_17_H_14_O_6_	98.93	−1.44	5.71	Pinobanksin acetate/Dimethyl kaempferol isomer 2	49 ± 3
37	23.70	285.0771	C_16_H_14_O_5_	99.62	−1.10	0.65	Isosakuranetin/5-O-methylpinobanksin	3.2 ± 0.2
38	23.80	249.1137	C_14_H_18_O_4_	98.77	−1.86	2.36	Isopentyl caffeate	14.0 ± 0.5
39	23.92	391.1195	C_23_H_20_O_6_	97.37	−2.11	0.59	Unknown	-
40	23.98	311.2232	C_18_H_32_O_4_	99.26	−1.45	0.61	Octadecenedioic acid	-
41	24.04	283.0616	C_16_H_12_O_5_	96.51	−1.94	0.78	Acacetin/Calycosin/Thevetiaflavona/ Biochanin A/Methyilgalangin/Pterosonin E/6-Methoxybaicalein isomer 2	3.5 ± 0.2
42	24.12	433.1298	C_25_H_22_O_7_	98.95	−1.21	1.44	Hydroxymethoxyphenylpropenyl pinobanksin	2.18 ± 0.05
43	24.40	295.0980	C_18_H_16_O_4_	99.29	−1.45	0.86	Cinnamyl caffeate	4.6 ± 0.2
44	24.68	231.1030	C_14_H_16_O_3_	98.62	−1.25	1.82	Drupanin/3-Prenyl-p-coumaric acid isomer 1	26 ± 2
45	24.97	231.1027	C_14_H_16_O_3_	98.41	−0.46	3.28	Drupanin/3-Prenyl-p -coumaric acid isomer 2	52 ± 4
46	25.48	327.0877	C_18_H_16_O_6_	99.43	−0.75	0.48	Pinobanksin-3-O-propionate	-
47	25.59	267.1028	C_17_H_16_O_3_	98.89	−0.68	0.78	Bencyl p-coumarate/Phenethyl p-coumarate	6.4 ± 0.3
48	26.14	263.1291	C_15_H_20_O_4_	98.32	−1.18	0.46	Abscisic acid	-
49	26.31	269.0822	C_16_H_14_O_4_	99.49	−1.00	1.32	Pinostrobin/Medicarpin/Alpinetin isomer 2	8.7 ± 0.5
50	26.81	271.0978	C_16_H_16_O_4_	99.51	−0.87	0.77	Vestitol/Neovestitol	3.2 ± 0.2
51	26.88	323.1293	C_20_H_20_O_4_	99.20	−1.27	0.89	Cinnamyl-3,4-dimethoxycinnamate	-
52	26.96	413.1976	C_24_H_30_O_6_	98.35	−1.52	1.06	Armillarin/Armillaripin	-
53	27.64	341.1035	C_19_H_18_O_6_	99.32	−1.26	0.33	Pinobanksin 3-O-butyrate isomer 2	1.17 ± 0.05
54	27.76	399.2182	C_24_H_32_O_5_	99.17	−1.14	0.38	Unknown	-
55	29.47	315.1605	C_19_H_24_O_4_	99.66	−0.84	0.30	Capillartemisin A/Gibberellin A9 isomer 1	14.0 ± 0.7
56	29.71	355.1191	C_20_H_20_O_6_	98.88	−1.00	0.60	Pinobanksin 3-O-pentanoate isomer 2	-
57	29.83	517.1873	C_30_H_30_O_8_	99.45	−0.93	0.31	Unknown	-
58	30.07	403.1191	C_24_H_20_O_6_	99.48	−0.88	0.34	Pinobanksin dihydrocinnamate	-
59	30.30	315.1604	C_19_H_24_O_4_	99.22	−0.78	2.88	Capillartemisin A/Gibberellin A9 isomer 2	-
60	30.97	315.1606	C_19_H_24_O_4_	99.31	−1.01	0.34	Capillartemisin A/Gibberellin A9 isomer 3	-
61	31.94	293.2127	C_18_H_30_O_3_	99.02	−1.68	0.89	Oxo-octadecadienoic acid	-
62	32.04	297.2440	C_18_H_34_O_3_	98.90	−1.52	0.32	Hydroxyoctadecanoic acid	-
63	32.39	293.2125	C_18_H_30_O_3_	99.18	−1.01	0.45	Oxo-octadecadienoic acid	-
64	33.02	299.1657	C_19_H_24_O_3_	99.29	−1.42	0.31	Artepillin C	-
65	33.30	565.3601	C_28_H_54_O_11_	98.33	−1.29	0.37	Unknown	-
66	33.92	565.3598	C_28_H_54_O_11_	99.29	−0.96	1.99	Unknown	-

(-) Indicating compounds not quantified.

**Table 2:** Results of characterization of PE microparticles (encapsulation efficiency of each phenolic compound).

Compounds	EE (%)			
	PE-IN	PE-IN/SA	PE-IN/P	PE-IN/CH
**Caffeic acid derivatives**				
Caffeoylglycerol	77.9 ± 2.4 bc	81.6 ± 1.1 c	71.5 ± 8.2 b	54.7 ± 10.9 a
Caffeic acid	83.2 ± 2.1 b	86.7 ± 0.5 b	72.1 ± 8.2 a	68.1 ± 6.3 a
Butyl caffeate	33.8 ± 8.9 bc	41.6 ± 2.4 c	30.0 ± 8.9 ab	23.4 ± 7.3 a
Prenyl caffeate isomer 1	48.7 ± 10.4 a	62.8 ± 4.9 b	43.2 ± 6.9 a	51.7 ± 3.6 ab
Prenyl caffeate isomer 2	49.2 ± 9.6 a	66.5 ± 3.2 b	42.4 ± 8.3 a	53.3 ± 5.6 a
β-Phenylethyl caffeate	50.5 ± 7.7 bc	57.2 ± 2.3 c	42.3 ± 3.5 ab	33.9 ± 8.4 a
Isopentyl caffeate	30.3 ± 6.7 a	46.0 ± 2.6 b	27.0 ± 5.6 a	19.8 ± 7.8 a
Cinnamyl caffeate	43.7 ± 4.4 b	44.9 ± 3.2 b	27.7 ± 2.8 a	32.0 ± 1.7 a
**Coumaric acid derivatives**				
p-Coumaric acid	86.7 ± 1.6 b	90.6 ± 0.6 b	75.5 ± 9.9 a	85.2 ± 1.9 b
Drupanin/3-Prenyl-p-coumaric acid isomer 1	48.4 ± 7.3 c	56.9 ± 3.7 c	34.0 ± 7.0 b	16.3 ± 5.6 a
Drupanin/3-Prenyl-p-coumaric acid isomer 2	49.6 ± 7.8 b	60.4 ± 3.2 b	35.6 ± 5.6 a	76.3 ± 3.6 c
Benzyl p-coumarate/Phenethyl p-coumarate	53.8 ± 6.5 b	53.7 ± 1.6 b	36.5 ± 7.2 a	50.8 ± 6.0 b
Capillartemisin A/Gibberellin A9	50.4 ± 5.7 c	49.9 ± 2.2 c	33.6 ± 13.9 b	18.0 ± 12.5 a
**Pinobanksin derivatives**				
Pinobanksin	45.4 ± 4.5 b	58.6 ± 1.9 c	47.1 ± 11.6 b	31.6 ± 11.4 a
Pinobanksin-3-O-pentanoate isomer 1	31.9 ± 6.5 a	45.4 ± 2.0 b	27.7 ± 6.0 a	34.9 ± 4.1 a
Pinobanksin acetate/Dimethyl kaempferol isomer 1	33.3 ± 7.0 ab	48.7 ± 2.6 c	25.4 ± 7.2 a	41.7 ± 3.9 bc
Pinobanksin acetate/Dimethyl kaempferol isomer 2	46.0 ± 6.7 b	54.8 ± 2.9 b	34.4 ± 7.9 a	32.7 ± 6.8 a
Isosakuranetin/5-O-Methylpinobanksin	35.4 ± 6.4 a	47.0 ± 1.7 b	28.3 ± 3.9 a	30.4 ± 4.4 a
Hydroxy methoxyphenylpropenyl pinobanksin/Pinocembrin-5-O-3-hydroxy-4-methoxyphenylpropionate	51.8 ± 3.8 b	47.2 ± 1.3 b	31.6 ± 5.7 a	28.8 ± 6.3 a
Pinobanksin-3-O-pentanoate isomer 2	50.2 ± 4.7 b	47.0 ± 1.3 b	31.1 ± 13.6 a	23.5 ± 7.5 a
**Other flavonoids**				
Rhamnetin/Methylquercetin isomer 1	39.5 ± 3.9 ab	46.9 ± 2.2 bc	36.0 ± 1,3 a	55.2 ± 7.1 c
Apigenin	49.9 ± 4.3 b	50.6 ± 3.2 b	34.5 ± 13.1 a	37.9 ± 5.2 a
Kaempferol	43.1 ± 4.3 b	46.9 ± 1.0 b	31.8 ± 4.8 ab	20.4 ± 9.6 a
Isokaempferide/Diosmetin	38.7 ± 7.3 ab	47.0 ± 1.8 b	31.3 ± 4.8 a	46.0 ± 6.7 b
Rhamnetin/Methylquercetin isomer 2	41.1 ± 4.6 b	40.6 ± 1.8 b	24.3 ± 4.6 a	58.1 ± 12.6 c
Dimethylquercetin/Eupaletin	40.3 ± 3.8 b	43.1 ± 1.4 bc	28.1 ± 12.9 a	49.5 ± 6.8 c
Pinostrobin/Medicarpin/Alpinetin isomer 1	42.1 ± 5.7 bc	50.8 ± 2.8 c	32.7 ± 6.1 ab	27.3 ± 9.3 a
Chrysin	53.0 ± 3.5 b	51.9 ± 2.5 b	32.6 ± 6.7 a	38.7 ± 5.1 a
Sakuranetin	41.1 ± 4.3 b	44.8 ± 2.9 b	28.3 ± 3.3 a	48.0 ± 6.9 b
Acacetin/Calycosin/Thevetiaflavone/Biochanin A/5-O-Methylgalangin/Pterosonin E/6-Methoxybaicalein isomer 1	54.2 ± 5.1 b	49.1 ± 0.9 b	30.4 ± 1.1 a	31.7 ± 4.7 a
Pinocembrin	42.2 ± 8.1 ab	48.8 ± 3.1 b	32.4 ± 6.7 a	38.3 ± 4.5 ab
Apigenin isomer	49.6 ± 5.2 bc	53.3 ± 2.5 c	31.3 ± 5.3 a	38.5 ± 3.9 ab
Kaempferide	44.8 ± 5.4 c	41.6 ± 1.5 bc	27.0 ± 13.8 a	35.3 ± 4.9 ab
Acacetin/Calycosin/Thevetiaflavone/Biochanin A/5-O-Methylgalangin/Pterosonin E/6-Methoxybaicalein isomer 2	49.3 ± 5.6 b	50.4 ± 2.7 b	31.4 ± 6.2 a	34.7 ± 4.3 a
Pinostrobin/Medicarpin/Alpinetin isomer 2	51.5 ± 5.2 c	33.4 ± 3.3 b	39.9 ± 14.7 b	21.9 ± 9.2 a
Vestitol/Neovestitol	46.1 ± 4.6 b	49.5 ± 3.2 b	30.7 ± 5.1 a	31.0 ± 4.3 a

Values: means ± standard deviations. Different letters represent levels of significance: *p*-value < 0.05.

**Table 3:** Results of characterization of PE microparticles (recovery of each phenolic compound).

Compounds	Recovery 1 (%)				Recovery 2 (%)			
	PE-IN	PE-IN/SA	PE-IN/P	PE-IN/CH	PE-IN	PE-IN/SA	PE-IN/P	PE-IN/CH
**Caffeic acid derivatives**								
Caffeoylglycerol	81.8 ± 6.2 c	40.9 ± 2.2 a	63.6 ± 14.6 b	63.8 ± 3.5 b	121.1 ± 8.6 c	70.0 ± 4.9 a	100.7 ± 6.7 b	96.5 ± 13.5 b
Caffeic acid	81.9 ± 5.2 c	40.3 ± 1.8 a	59.9 ± 14.6 b	80.6 ± 7.7 c	129.8 ± 13.6 c	74.6 ± 8.6 a	100.5 ± 9.1 b	101.1 ± 8.2 b
Butyl caffeate	83.8 ± 5.3 c	34.5 ± 2.3 a	58.6 ± 15.7 b	69.2 ± 5.5 b	61.6 ± 4.6 a	59.6 ± 5.7 a	64.4 ± 6.8 a	81.9 ± 7.2 b
Prenyl caffeate isomer 1	67.1 ± 5.8 c	29.4 ± 2.2 a	40.2 ± 14.9 b	65.2 ± 7.7 c	32.4 ± 4.9 a	61.9 ± 8.8 b	41.4 ± 7.8 a	79.9 ± 10.7 c
Prenyl caffeate isomer 2	66.7 ± 6.7 b	41.5 ± 3.5 a	35.0 ± 14.4 a	87.8 ± 13.8 c	26.8 ± 3.8 a	68.1 ± 11.1 b	37.6 ± 7.6 a	85.9 ± 9.7 b
β-Phenylethyl caffeate	87.5 ± 7.9 d	30.6 ± 1.9 a	48.5 ± 15.2 b	63.0 ± 4.9 c	32.4 ± 13.2 a	60.0 ± 7.6 b	44.3 ± 5.7 a	76.9 ± 5.6 c
Isopentyl caffeate	88.3 ± 6.9 c	32.0 ± 1.3 a	54.4 ± 14.4 b	61.9 ± 5.8 b	43.4 ± 3.4 a	58.4 ± 4.8 b	51.1 ± 5.5 ab	76.2 ± 6.6 c
Cinnamyl caffeate	104.1 ± 8.9 c	36.1 ± 2.6 a	61.1 ± 13.4 b	52.9 ± 9.6 b	41.9 ± 3.0 a	56.1 ± 4.9 b	51.8 ± 3.1 ab	73.9 ± 9.8 c
**Coumaric acid derivatives**								
p-Coumaric acid	76.3 ± 5.9 c	38.6 ± 1.3 a	56.9 ± 18.3 b	99.6 ± 11.9 d	115.9 ± 12.9 b	76.1 ± 8.6 a	95.9 ± 10.8 ab	99.3 ± 9.4 ab
Drupanin/3-Prenyl-p-coumaric acid isomer 1	74.0 ± 7.1 c	26.6 ± 2.8 a	40.8 ± 13.4 b	35.5 ± 4.6 ab	23.9 ± 2.9 a	56.4 ± 9.0 c	37.0 ± 4.7 b	70.6 ± 5.2 d
Drupanin/3-Prenyl-p-coumaric acid isomer 2	74.7 ± 7.8 b	26.9 ± 2.4 a	39.8 ± 14.0 a	139.6 ± 22.3 c	21.2 ± 12.5 a	56.9 ± 8.7 b	34.6 ± 4.6 a	70.5 ± 6.8 c
Benzyl p-coumarate/Phenethyl p-coumarate	91.7 ± 9.1 d	31.2 ± 2.9 a	48.0 ± 14.9 b	76.0 ± 10.5 c	25.0 ± 2.4 a	56.3 ± 7.5 c	39.7 ± 4.9 b	72.0 ± 14.2 d
Capillartemisin A/Gibberellin A9	128.0 ± 13.3 c	35.7 ± 3.2 a	62.6 ± 15.8 b	56.0 ± 7.2 b	31.9 ± 3.2 a	54.5 ± 6.2 b	43.4 ± 3.8 b	70.9 ± 4.9 c
**Pinobanksin derivatives**								
Pinobanksin	77.1 ± 4.7 c	34.5 ± 2.3 a	53.7 ± 16.7 b	59.4 ± 7.0 b	61.6 ± 3.5 a	65.0 ± 5.7 a	65.8 ± 10.0 a	87.2 ± 5.0 b
Pinobanksin-3-O-pentanoate isomer 1	93.8 ± 9.9 d	37.1 ± 2.3 b	60.4 ± 15.7 c	28.5 ± 25.3 a	57.3 ± 4.2 a	62.0 ± 5.1 a	60.1 ± 7.2 a	81.5 ± 7.5 b
Pinobanksin acetate/Dimethyl kaempferol isomer 1	91.2 ± 8.6 c	38.6 ± 1.6 a	56.2 ± 14.8 b	82.2 ± 5.4 c	45.0 ± 3.2 a	66.1 ± 5.1 b	52.8 ± 5.4 a	104.3 ± 12.7 c
Pinobanksin acetate/Dimethyl kaempferol isomer 2	85.2 ± 8.2 d	30.2 ± 2.0 a	44.6 ± 15.4 b	61.5 ± 6.1 c	28.1 ± 2.7 a	58.8 ± 7.4 b	39.7 ± 4.9 a	73.1 ± 7.0 b
Isosakuranetin/5-O-Methylpinobanksin	85.7 ± 8.5 d	32.1 ± 1.8 a	53.3 ± 12.6 b	66.1 ± 4.8 c	39.7 ± 3.2 a	58.0 ± 5.8 b	50.1 ± 4.5 ab	73.7 ± 6.4 c
Hydroxy methoxyphenylpropenyl pinobanksin/Pinocembrin-5-O-3-hydroxy-4-methoxyphenylpropionate	112.0 ± 9.1 c	35.9 ± 2.6 a	58.8 ± 15.5 b	62.8 ± 5.0 b	38.4 ± 2.0 a	56.6 ± 4.6 b	47.3 ± 2.7 ab	75.1 ± 10.1 c
Pinobanksin-3-O-pentanoate isomer 2	121.4 ± 10.6 c	37.8 ± 3.1 a	62.4 ± 14.1 b	60.8 ± 7.9 b	34.8 ± 2.5 a	55.9 ± 5.3 b	47.3 ± 2.6 b	72.5 ± 5.6 c
**Other flavonoids**								
Rhamnetin/Methylquercetin isomer 1	91.5 ± 7.1 c	37.4 ± 2.0 a	62.1 ± 14.2 b	100.8 ± 11.7 c	72.5 ± 4.9 ab	60.8 ± 4.3 a	67.4 ± 6.5 a	85.3 ± 11.7 b
Apigenin	89.5 ± 9.7 d	33.0 ± 2.4 a	51.9 ± 15.0 b	68.7 ± 4.9 c	43.3 ± 3.7 a	58.0 ± 5.7 b	48.1 ± 4.3 ab	77.8 ± 11.5 c
Kaempferol	90.9 ± 8.3 c	35.1 ± 2.1 a	55.8 ± 14.2 b	56.0 ± 10.1 b	54.9 ± 3.8 a	57.1 ± 4.7 a	54.7 ± 4.5 a	81.2 ± 6.6 b
Isokaempferide/Diosmetin	92.1 ± 7.2 c	36.0 ± 2.5 a	57.9 ± 15.0 b	88.7 ± 11.0 c	50.3 ± 3.6 a	58.5 ± 5.0 a	53.9 ± 5.3 a	78.4 ± 7.7 b
Rhamnetin/Methylquercetin isomer 2	97.2 ± 9.2 c	34.0 ± 2.4 a	56.4 ± 14.3 b	106.0 ± 19.4 c	46.0 ± 3.4 a	53.9 ± 5.2 a	49.8 ± 3.3 a	74.5 ± 7.6 b
Dimethylquercetin/Eupaletin	96.2 ± 8.5 d	34.9 ± 1.7 a	58.1 ± 14.8 b	84.1 ± 6.6 c	44.1 ± 2.7 a	55.8 ± 4.2 b	51.4 ± 3.6 ab	75.9 ± 6.7 c
Pinostrobin/Medicarpin/Alpinetin isomer 1	87.5 ± 9.9 c	32.3 ± 2.3 a	52.5 ± 16.1 b	57.4 ± 5.7 b	38.1 ± 3.0 a	59.9 ± 5.0 b	48.7 ± 5.8 ab	77.5 ± 7.6 c
Chrysin	92.7 ± 7.1 d	32.8 ± 2.5 a	48.7 ± 13.9 b	71.5 ± 7.8 c	29.5 ± 12.1 a	57.3 ± 5.6 b	41.4 ± 3.2 a	75.3 ± 5.5 c
Sakuranetin	94.0 ± 6.0 c	34.9 ± 2.6 a	53.1 ± 13.3 b	93.4 ± 11.8 c	39.9 ± 2.8 a	57.3 ± 4.8 b	49.4 ± 3.7 ab	77.0 ± 7.6 c
Acacetin/Calycosin/Thevetiaflavone/Biochanin A/5-O-Methylgalangin/Pterosonin E/6-Methoxybaicalein isomer 1	97.8 ± 10.9 c	32.6 ± 2.3 a	53.6 ± 15.4 b	64.2 ± 5.4 b	31.1 ± 2.6 a	54.0 ± 5.3 b	42.8 ± 3.6 ab	72.2 ± 3.6 c
Pinocembrin	83.2 ± 9.0 c	34.0 ± 1.4 a	50.3 ± 14.9 b	76.0 ± 7.8 c	34.7 ± 3.3 a	57.8 ± 6.4 b	47.2 ± 5.5 ab	75.1 ± 7.3 c
Apigenin isomer	88.1 ± 9.3 d	31.5 ± 1.9 a	47.0 ± 15.9 b	60.7 ± 5.9 c	26.6 ± 12.1 a	56.5 ± 4.9 b	39.2 ± 4.3 a	73.5 ± 4.9 c
Kaempferide	105.5 ± 9.4 d	36.7 ± 2.1 a	59.9 ± 13.4 b	79.8 ± 6.0 c	42.8 ± 2.9 a	54.5 ± 4.7 b	50.3 ± 2.7 ab	74.7 ± 7.6 c
Acacetin/Calycosin/Thevetiaflavone/Biochanin A/5-O-Methylgalangin/Pterosonin E/6-Methoxybaicalein isomer 2	93.5 ± 10.1 d	33.3 ± 3.3 a	51.4 ± 15.1 b	66.5 ± 4.7 c	32.0 ± 5.2 a	57.7 ± 7.5 b	44.0 ± 6.0 a	75.1 ± 3.4 c
Pinostrobin/Medicarpin/Alpinetin isomer 2	101.3 ± 18.1 c	21.6 ± 3.5 a	50.9 ± 16.0 b	50.2 ± 6.1 b	24.8 ± 2.4 a	36.2 ± 6.2 ab	39.3 ± 3.0 b	63.4 ± 3.2 c
Vestitol/Neovestitol	92.0 ± 10.2 d	32.5 ± 2.6 a	52.2 ± 13.7 b	63.7 ± 4.5 c	29.9 ± 2.5 a	55.3 ± 5.0 c	42.8 ± 2.3 b	69.1 ± 5.3 d

Values: means ± standard deviations. Different letters represent the level of significance: *p* < 0.05.

**Table 4:** Results of characterization of the microparticles (total phenolic content, total flavonoid content, and antioxidant capacity).

Sample	TPC (mg GAE */g)	TFC (mg QE **/g)	FRAP (µmol TE ***/g)	ORAC (µmol TE ***/g)
PE-IN	7.1 ± 0.2 a	0.42 ± 0.02 a	30 ± 1 a	230 ± 10 a
PE-IN/SA	10.9 ± 0.6 c	0.90 ± 0.07 c	38 ± 2 b	340 ± 20 c
PE-IN/P	10.4 ± 0.2 c	0.87 ± 0.05 c	40 ± 3 b	340 ± 30 c
PE-IN/CH	9.8 ±0.6 b	0.75 ± 0.07 b	31 ± 2 a	260 ± 10 b

Values: means ± standard deviations. Different letters represent the level of significance: *p* < 0.05. * GAE: gallic acid equivalent; ** QE: quercetin equivalent; *** TE: Trolox equivalent.

**Table 5:** Results of recovery percentage under gastric digestion.

Compounds	PE (Rec%)	PE-IN (Rec%)	PE-IN/SA (Rec%)	PE-IN/P (Rec%)	PE-IN/CH (Rec%)
**Caffeic acid derivatives**					
Caffeoylglycerol	325.4 ± 12.4 d	57.8 ± 1.5 c	38.3 ± 1.9 b	63.8 ± 0.9 c	14.4 ± 0.3 a
Caffeic acid	272.0 ± 14.5 e	48.0 ± 0.5 c	33.1 ± 0.5 b	58.7 ± 0.8 d	12.5 ± 0.4 a
Butyl caffeate	65.0 ± 2.6 e	20.0 ± 0.6 c	7.7 ± 0.0 b	25.1 ± 0.4 d	5.3 ± 0.1 a
Prenyl caffeate isomer 1	16.1 ± 0.5 d	13.4 ± 0.2 c	2.3 ± 0,1 b	18.9 ± 0.2 e	1.7 ± 0.0 a
Prenyl caffeate isomer 2	10.7 ± 0.3 b	11.0 ± 0.3 b	1.4 ± 0.0 a	18.6 ± 0.3 c	1.3 ± 0.0 a
β-Phenylethyl caffeate	18.1 ± 0.1 d	9.5 ± 0.1 c	1.9 ± 0.0 a	20.3 ± 0.5 e	3.0 ± 0.1 b
Isopentyl caffeate	40.1 ± 0.9 d	15.2 ± 0.4 b	4.3 ± 0.1 a	25.9 ± 0.5 c	4.6 ± 0.1 a
Cinnamyl caffeate	28.4 ± 8.5 b	6.8 ± 1.3 a	2.1 ± 1.1 a	29.9 ± 1.0 b	6.4 ± 3.1 a
**Coumaric acid derivatives**					
p-Coumaric acid	117.5 ± 5.2 e	41.8 ± 1.8 c	25.4 ± 1.7 b	51.7 ± 2.1 d	7.0 ± 0.1 a
Drupanin/3-Prenyl-p-coumaric acid isomer 1	5.8 ± 0.2 d	5.3 ± 0.1 c	0.9 ± 0.0 a	14.9 ± 0.6 e	1.4 ± 0.1 b
Drupanin/3-Prenyl-p-coumaric acid isomer 2	4.6 ± 0.1 c	4.7 ± 0.0 c	0.7 ± 0.0 a	15.1 ± 0.3 d	1.2 ± 0.0 b
Benzyl p-coumarate/Phenethyl p-coumarate	2.4 ± 0.2 d	2.1 ± 0.3 c	0.4 ± 0.0 a	14.2 ± 0.2 e	1.5 ± 0.1 b
Capillartemisin A/Gibberellin A9	8.3 ± 0.6 d	2.5 ± 0.1 b	0.6 ± 0.0 a	25.2 ± 0.3 e	4.6 ± 0.1 c
**Pinobanksin derivatives**					
Pinobanksin	93.3 ± 3.1 e	49.5 ± 1.2 c	17.1 ± 0.3 b	55.2 ± 1.0 d	7.7 ± 0.1 a
Pinobanksin-3-O-pentanoate isomer 1	142.6 ± 30.2 d	51.7 ± 0.7 b	15.6 ± 0.3 a	89.4 ± 5.8 c	15.6 ± 0.7 a
Pinobanksin acetate/Dimethyl kaempferol isomer 1	39.5 ± 1.3 d	16.4 ± 0.2 b	4.0 ± 0.1 a	28.4 ± 0.7 c	3.9 ± 0.2 a
Pinobanksin acetate/Dimethyl kaempferol isomer 2	11.0 ± 0.4 d	9.0 ± 0.1 c	1.4 ± 0.0 a	22.8 ± 0.6 e	2.3 ± 0.1 b
Isosakuranetin/5-O-Methylpinobanksin	30.3 ± 0.7 d	13.4 ± 0.4 b	3.4 ± 0.1 a	21.9 ± 0.6 c	4.0 ± 0.2 a
Hydroxy methoxyphenylpropenyl pinobanksin/Pinocembrin-5-O-3-hydroxy-4-methoxyphenylpropionate	4.7 ± 1.0 c	3.1 ± 0.3 b	0.8 ± 0.1 a	23.8 ± 0.6 d	4.0 ± 0.1 c
Pinobanksin-3-O-pentanoate isomer 2	16.3 ± 6.5 b	3.2 ± 1.4 a	1.0 ± 0.9 a	26.1 ± 0.7 c	4.9 ± 2.1 a
**Other flavonoids**					
Rhamnetin/Methylquercetin isomer 1	249.4 ± 6.9 e	74.4 ± 0.5 c	27.9 ± 0.4 b	93.7 ± 3.1 d	19.1 ± 0.3 a
Apigenin	25.3 ± 1.7 c	14.7 ± 0.3 b	3.3 ± 0.1 a	30.6 ± 0.9 d	4.2 ± 0.1 a
Kaempferol	61.7 ± 1.2 d	25.4 ± 0.7 b	7.9 ± 0.1 a	41.5 ± 1.3 c	7.3 ± 0.3 a
Isokaempferide/Diosmetin	42.2 ± 0.7 d	16.8 ± 0.5 b	4.7 ± 0.1 a	28.5 ± 0.5 c	5.2 ± 0.1 a
Rhamnetin/Methylquercetin isomer 2	63.4 ± 0.5 e	25.5 ± 0.8 c	7.7 ± 0.2 a	60.1 ± 3.1 d	11.5 ± 0.4 b
Dimethylquercetin/Eupaletin	57.1 ± 2.0 d	22.8 ± 0.3 c	6.3 ± 0.1 a	59.2 ± 3.3 d	10.8 ± 0.4 b
Pinostrobin/Medicarpin/Alpinetin isomer 1	26.0 ± 0.7 e	12.9 ± 0.4 c	3.0 ± 0.0 a	23.2 ± 0.5 d	3.7 ± 0.1 b
Chrysin	5.2 ± 0.4 d	3.7 ± 0.1 c	0.7 ± 0.0 a	20.4 ± 0.7 e	2.3 ± 0.0 b
Sakuranetin	16.6 ± 0.7 d	6.9 ± 0.1 c	1.8 ± 0.1 a	16.9 ± 0.6 d	3.0 ± 0.1 b
Acacetin/Calycosin/Thevetiaflavone/Biochanin A/5-O-Methylgalangin/Pterosonin E/6-Methoxybaicalein isomer 1	3.4 ± 0.3 c	2.3 ± 0.1 b	0.5 ± 0.0 a	18.8 ± 0.2 d	2.4 ± 0.1 b
Pinocembrin	15.3 ± 0.1 d	9.8 ± 0.2 c	2.1 ± 0.1 a	20.8 ± 0.7 e	2.7 ± 0.1 b
Apigenin isomer	7.3 ± 0.2 d	6.7 ± 0.1 c	1.1 ± 0.0 a	28.1 ± 0.9 e	3.1 ± 0.1 b
Kaempferide	15.4 ± 1.1 d	5.2 ± 0.2 c	1.5 ± 0.1 a	21.4 ± 0.5 e	4.3 ± 0.2 b
Acacetin/Calycosin/Thevetiaflavone/Biochanin A/5-O-Methylgalangin/Pterosonin E/6-Methoxybaicalein isomer 2	7.7 ± 0.4 d	4.6 ± 0.2 c	1.0 ± 0.0 a	20.5 ± 0.7 e	2.7 ± 0.1 b
Pinostrobin/Medicarpin/Alpinetin isomer 2	4.4 ± 0.4 c	3.0 ± 0.1 b	0.8 ± 0.0 a	20.0 ± 0.8 d	2.8 ± 0.1 b
Vestitol/Neovestitol	7.9 ± 0.6 d	4.0 ± 0.1 c	0.9 ± 0.1 a	20.0 ± 0.6 e	3.1 ± 0.1 b

Values: means ± standard deviations. Different letters represent the level of significance: *p* < 0.05.

**Table 6:** Results of recovery and final bioaccessibility percentage under intestinal digestion for PE, PE-IN, and PE-IN/SA.

Compounds	PE (Rec%)			PE-IN (Rec%)			PE-IN/SA (Rec%)		
	30 min	60 min	120 min	30 min	60 min	120 min	30 min	60 min	120 min
**Caffeic acid derivatives**									
Caffeoylglycerol	164.8 ± 2.6 eB	145.9 ± 1.5 eA	147.5 ± 7.6 eA	69.0 ± 1.4 bB	63.8 ± 0,2 bA	58.2 ± 1.1 bA	88.3 ± 3.5 dB	87.7 ± 2.0 dA	89.7 ± 2.1 dA
Caffeic acid	183.1 ± 2.4 eA	198.2 ± 5.0 eA	230.7 ± 18.6 eB	68.0 ± 2.1 bA	71.9 ± 1.9 bA	78.6 ± 3.6 bB	100.3 ± 1.2 dA	92.1 ± 0.9 dA	101.9 ± 1.3 dB
Butyl caffeate	38.4 ± 1.2 dB	37.9 ± 1.2 dA	40.7 ± 3.3 dB	28.0 ± 0.5 cB	26.9 ± 0.7 cA	27.9 ± 0.8 cB	42.1 ± 0.8 eB	39.3 ± 1.7 eA	42.9 ± 1.1 eB
Prenyl caffeate isomer 1	10.0 ± 0.3 bB	9.9 ± 0.2 bA	11.1 ± 0.9 bB	22.2 ± 0.6 eB	21.1 ± 0.8 eA	22.7 ± 0.5 eB	16.0 ± 1.0 cB	14.4 ± 0.5 cA	16.2 ± 0.3 cB
Prenyl caffeate isomer 2	6.6 ± 0.1 bB	5.9 ± 0.1 bA	6.0 ± 0.4 bA	19.6 ± 0.6 eB	17.9 ± 0.6 eA	17.5 ± 0.6 eA	11.7 ± 0.7 cB	10.5 ± 0.4 cA	11.4 ± 0.3 cA
β-Phenylethyl caffeate	15.5 ± 0.5 bA	15.3 ± 0.5 bA	16.5 ± 1.1 bA	21.3 ± 0.7 dA	21.1 ± 0.5 dA	22.4 ± 0.7 dA	18.1 ± 1.1 cA	17.2 ± 0.6 cA	18.0 ± 0.6 cA
Isopentyl caffeate	26.6 ± 0.8 cB	25.2 ± 1.5 cA	25.4 ± 1.2 cA	26.3 ± 0.9 cB	26.0 ± 0.1 cA	26.0 ± 0.6 cA	33.8 ± 1.6 dB	31.9 ± 0.7 dA	31.9 ± 0.4 dA
Cinnamyl caffeate	29.4 ± 1.0 eC	27.1 ± 0.3 eB	25.0 ± 0.9 eA	25.0 ± 0.4 cC	23.7 ± 0.5 cB	24.1 ± 0.5 cA	26.8 ± 1.1 dC	25.5 ± 0.7 dB	25.2 ± 0.5 dA
**Coumaric acid derivatives**									
p-Coumaric acid	93.0 ± 3.8 eA	90.0 ± 3.1 eA	104.3 ± 14.7 eB	62.6 ± 1.6 cA	64.0 ± 2.5 cA	66.4 ± 3.7 cB	55.0 ± 2.3 bA	53.5 ± 0.8 bA	55.3 ± 2.3 bB
Drupanin/3-Prenyl-p-coumaric acid isomer 1	3.5 ± 0.1 bA	3.2 ± 0.2 bA	2.7 ± 0.2 bA	9.4 ± 0.3 dA	9.0 ± 0.3 dA	9.6 ± 0.3 dA	9.6 ± 0.8 dA	9.0 ± 0.5 dA	9.6 ± 0.5 dA
Drupanin/3-Prenyl-p-coumaric acid isomer 2	3.6 ± 0.1 bC	2.7 ± 0.1 bB	2.2 ± 0.1 bA	11.9 ± 0.4 dC	9.2 ± 0.2 dB	8.2 ± 0.2 dA	9.4 ± 0.5 cC	8.6 ± 0.2 cB	8.7 ± 0.2 cA
Benzyl p-coumarate/Phenethyl p-coumarate	3.4 ± 0.2 bA	3.5 ± 0.2 bA	2.9 ± 0.5 bA	8.3 ± 0.5 eA	8.0 ± 0.3 eA	9.0 ± 0.2 eA	6.1 ± 0.4 cA	5.9 ± 0.5 cA	6.1 ± 0.3 cA
Capillartemisin A/Gibberellin A9	18.6 ± 0.4 cA	19.4 ± 1.0 cA	18.4 ± 0.8 cA	25.3 ± 0.7 eA	25.4 ± 0.3 eA	27.2 ± 0.7 eA	17.7 ± 0.4 A	17.6 ± 0.3 bA	17.9 ± 0.7 bA
**Pinobanksin derivatives**									
Pinobanksin	76.7 ± 4.0 bA	77.8 ± 2.8 bA	83.9 ± 10.9 bA	87.3 ± 2.0 cA	92.1 ± 0.7 cA	93.2 ± 2.2 cA	80.6 ± 1.2 bA	73.8 ± 2.5 bA	76.0 ± 2.2 bA
Pinobanksin-3-O-pentanoate isomer 1	49.0 ± 5.5 bC	40.0 ± 6.6 bB	32.4 ± 8.0 bA	53.7 ± 0.9 cC	50.8 ± 0.5 cB	46.0 ± 3.1 cA	92.9 ± 10.2 eC	84.5 ± 5.6 eB	86.1 ± 3.9 eA
Pinobanksin acetate/Dimethyl kaempferol isomer 1	45.9 ± 1.9 bC	39.5 ± 0.8 bA	42.1 ± 3.2 bB	50.7 ± 3.1 dC	45.2 ± 0.8 dA	49.4 ± 1.0 dB	52.0 ± 3.2 dC	48.5 ± 0.8 dA	47.7 ± 1.1 dB
Pinobanksin acetate/Dimethyl kaempferol isomer 2	19.1 ± 1.6 bA	19.7 ± 0.6 bA	20.6 ± 2.1 bA	44.4 ± 1.7 eA	45.0 ± 1.1 eA	46.2 ± 1.6 eA	33.8 ± 0.8 cA	31.9 ± 1.2 cA	30.7 ± 0.7 cA
Isosakuranetin/5-O-Methylpinobanksin	21.7 ± 0.5 cB	20.7 ± 0.2 cB	19.3 ± 0.4 cA	22.9 ± 0.8 dB	23.3 ± 0.2 dB	22.4 ± 0.8 dA	29.9 ± 1.6 eB	27.7 ± 0.5 eB	27.7 ± 0.6 eA
Hydroxy methoxyphenylpropenyl pinobanksin/Pinocembrin-5-O-3-hydroxy-4-methoxyphenylpropionate	15.2 ± 0.4 cA	16.4 ± 0.9 cA	15.8 ± 0.7 cA	18.0 ± 0.5 dA	18.6 ± 0.4 dA	19.7 ± 0.3 dA	13.0 ± 0.8 bA	12.8 ± 0.7 bA	11.9 ± 0.3 bA
Pinobanksin-3-O-pentanoate isomer 2	22.7 ± 0.9 cA	24.4 ± 1.6 cA	23.7 ± 1.5 cA	24.9 ± 0.8 dA	25.3 ± 0.5 dA	27.4 ± 0.8 dA	31.1 ± 4.0 eA	32.2 ± 0.5 eA	27.5 ± 1.8 eA
**Other flavonoids**									
Rhamnetin/Methylquercetin isomer 1	156.7 ± 9.4 dA	157.0 ± 5.7 dA	168.9 ± 14.3 dA	86.7 ± 4.3 bA	88.9 ± 1.1 bA	93.5 ± 1.9 bA	146.6 ± 5.1 cA	139.5 ± 4.1 cA	132.4 ± 1.6 cA
Apigenin	28.5 ± 2.2 bA	31.5 ± 2.6 bA	34.8 ± 4.6 bA	33.1 ± 1.2 cA	36.0 ± 1.2 cA	38.5 ± 0.9 cA	38.4 ± 2.6 cA	37.9 ± 0.4 cA	33.3 ± 1.6 cA
Kaempferol	23.8 ± 3.9 bB	20.1 ± 7.5 bB	11.8 ± 6.4 bA	28.7 ± 1.3 cB	27.0 ± 1.3 cB	23.2 ± 1.1 cA	52.6 ± 2.2 eB	52.9 ± 1.6 eB	50.3 ± 1.4 eA
Isokaempferide/Diosmetin	39.0 ± 1.7 dA	41.5 ± 1.5 dA	44.1 ± 4.2 dA	30.5 ± 1.0 cA	32.7 ± 0.1 cA	35.4 ± 0.9 cA	43.1 ± 1.0 dA	42.8 ± 0.5 dA	39.6 ± 1.2 dA
Rhamnetin/Methylquercetin isomer 2	9.8 ± 2.6 aB	7.9 ± 2.7 aB	5.7 ± 2.1 aA	16.0 ± 2.9 bB	13.7 ± 1.9 bB	10.1 ± 0.2 bA	44.3 ± 9.3 dB	43.8 ± 4.8 dB	42.8 ± 1.7 dA
Dimethylquercetin/Eupaletin	55.6 ± 3.2 dB	55.2 ± 2.2 dAB	57.8 ± 3.0 dA	51.2 ± 2.1 cB	51.2 ± 0.9 cAB	54.7 ± 1.3 cA	56.6 ± 1.3 cB	53.5 ± 1.0 cAB	49.9 ± 0.8 cA
Pinostrobin/Medicarpin/Alpinetin isomer 1	16.6 ± 0.5 bC	13.1 ± 0.2 bB	11.1 ± 0.4 bA	22.0 ± 0.4 cC	18.0 ± 0.4 cB	16.4 ± 0.4 cA	25.0 ± 1.2 dC	24.3 ± 0.5 dB	23.8 ± 0.9 dA
Chrysin	10.3 ± 0.6 bA	11.6 ± 0.6 bB	12.6 ± 1.5 bA	18.9 ± 0.2 bA	20.2 ± 0.9 bB	22.6 ± 0.6 bA	16.5 ± 2.2 bA	17.4 ± 0.4 bB	14.5 ± 1.3 bA
Sakuranetin	16.6 ± 0.5 cA	17.2 ± 0.4 cA	16.9 ± 1.1 cA	16.7 ± 0.2 cA	17.0 ± 0.4 cA	17.8 ± 0.4 cA	18.5 ± 0.7 dA	18.0 ± 0.3 dA	17.5 ± 0.4 dA
Acacetin/Calycosin/Thevetiaflavone/Biochanin A/5-O-Methylgalangin/Pterosonin E/6-Methoxybaicalein isomer 1	8.4 ± 0.3 bA	8.8 ± 0.4 bA	8.7 ± 0.7 bA	14.6 ± 0.6 dA	14.9 ± 0.3 dA	16.1 ± 0.1 dA	8.7 ± 0.1 bA	8.2 ± 0.1 bA	7.8 ± 0.1 bA
Pinocembrin	20.9 ± 2.0 bA	23.0 ± 1.3 bAB	25.1 ± 3.2 bB	33.6 ± 2.2 dA	36.0 ± 0.9 dAB	39.3 ± 1.1 dB	37.5 ± 0.6 dA	35.3 ± 0.8 dAB	33.8 ± 1.6 dB
Apigenin isomer	10.1 ± 0.3 bB	10.7 ± 0.1 bB	9.0 ± 0.5 bA	25.6 ± 0.7 dB	26.0 ± 0.5 dB	26.9 ± 0.5 dA	21.1 ± 1.8 cB	21.3 ± 0.1 cB	18.3 ± 1.4 cA
Kaempferide	15.4 ± 1.0 bB	15.9 ± 0.8 bB	14.3 ± 0.9 bA	15.5 ± 0.1 bB	15.6 ± 0.1 bB	16.2 ± 0.2 bA	17.1 ± 0.2 cB	16.8 ± 0.4 cB	16.3 ± 0.4 cA
Acacetin/Calycosin/Thevetiaflavone/Biochanin A/5-O-Methylgalangin/Pterosonin E/6-Methoxybaicalein isomer 2	13.6 ± 0.9 bA	15.0 ± 1.0 bA	16.3 ± 2.4 bA	21.3 ± 0.7 eA	22.3 ± 0.5 eA	25.7 ± 0.7 eA	22.1 ± 3.0 dA	22.5 ± 0.7 dA	19.0 ± 1.1 dA
Pinostrobin/Medicarpin/Alpinetin isomer 2	0.3 ± 0.1 aB	0.3 ± 0.1 aAB	0.4 ± 0.1 aA	0.3 ± 0.1 aB	0.3 ± 0.1 aAB	0.3 ± 0.1 aA	0.5 ± 0.1 aB	0.4 ± 0.1 aAB	0.3 ± 0.1 aA
Vestitol/Neovestitol	10.4 ± 0.7 bB	10.1 ± 1.1 bB	7.9 ± 0.3 bA	14.9 ± 0.3 eB	14.8 ± 0.2 eB	14.6 ± 0.5 eA	14.5 ± 0.3 dB	14.6 ± 0.5 dB	13.1 ± 0.2 dA

Values: means ± standard deviations. Different letters represent the level of significance: *p* < 0.05. Lowercase letters indicate differences between treatments and capital letters indicate differences between digestion times.

**Table 7:** Results of recovery and final bioaccessibility percentage under intestinal digestion for PE-IN/P and PE-IN/CH.

Compounds	PE-IN/P (Rec%)			PE-IN/CH (Rec%)		
	30 min	60 min	120 min	30 min	60 min	120 min
**Caffeic acid derivatives**						
Caffeoylglycerol	77.2 ± 4.8 cB	73.4 ± 2.1 cA	70.3 ± 2.1 cA	19.0 ± 1.2 aB	16.9 ± 0.5 aA	15.7 ± 0.3 aA
Caffeic acid	78.2 ± 2.0 cA	83.1 ± 1.5 cB	84.1 ± 2.8 cB	20.1 ± 0.8 aA	20.6 ± 0.5 aA	21.5 ± 0.3 aB
Butyl caffeate	26.7 ± 0.9 bB	26.2 ± 0.8 bA	24.9 ± 0.8 bB	5.1 ± 0.7 aB	4.4 ± 0.5 aA	4.3 ± 0.2 aB
Prenyl caffeate isomer 1	19.9 ± 1.9 dB	18.9 ± 1.1 dA	17.6 ± 1.0 dB	1.5 ± 0.3 aB	1.3 ± 0.2 aA	1.1 ± 0.1 aB
Prenyl caffeate isomer 2	17.7 ± 2.2 dB	16.4 ± 1.1 dA	14.5 ± 1.3 dA	1.0 ± 0.3 B	0.7 ± 0.2 aA	0.6 ± 0.1 aA
β-Phenylethyl caffeate	17.9 ± 1.4 cA	17.2 ± 1.2 cA	15.9 ± 1.0 cA	2.2 ± 0.7 aA	1.8 ± 0.5 aA	1.4 ± 0.4 aA
Isopentyl caffeate	24.3 ± 2.2 bB	23.3 ± 1.1 bA	21.4 ± 1.3 bA	3.5 ± 0.9 aB	2.9 ± 0.5 aA	2.5 ± 0.4 aA
Cinnamyl caffeate	24.7 ± 2.5 bC	23.2 ± 2.0 bB	21.0 ± 1.7 bA	4.4 ± 1.4 aC	3.3 ± 1.2 aB	2.0 ± 1.4 aA
**Coumaric acid derivatives**						
p-Coumaric acid	74.8 ± 3.1 dA	75.2 ± 2.3 dA	75.4 ± 0.9 dB	12.8 ± 0.6 aA	12.0 ± 0.3 aA	12.3 ± 0.5 aB
Drupanin/3-Prenyl-p-coumaric acid isomer 1	9.0 ± 1.3 cA	8.6 ± 1.1 cA	8.6 ± 1.0 cA	1.0 ± 0.3 aA	0.7 ± 0.2 aA	0.5 ± 0.1 aA
Drupanin/3-Prenyl-p-coumaric acid isomer 2	11.1 ± 1.5 dC	9.6 ± 1.2 dB	8.6 ± 0.9 dA	0.8 ± 0.3 aC	0.5 ± 0.2 aB	0.4 ± 0.1 aA
Benzyl p-coumarate/Phenethyl p-coumarate	7.6 ± 1.3 dA	7.2 ± 1.1 dA	6.8 ± 0.7 dA	0.9 ± 0.4 aA	0.6 ± 0.2 aA	0.4 ± 0.2 aA
Capillartemisin A/Gibberellin A9	21.3 ± 2.2 dA	20.3 ± 1.7 dA	19.1 ± 0.8 dA	2.9 ± 1.1 aA	2.2 ± 0.8 aA	1.3 ± 0.8 aA
**Pinobanksin derivatives**						
Pinobanksin	87.2 ± 2.4 cA	90.2 ± 3.1 cA	93.7 ± 0.6 cA	14.0 ± 2.0 aA	13.2 ± 1.7 aA	12.0 ± 1.0 aA
Pinobanksin-3-O-pentanoate isomer 1	57.0 ± 5.0 dC	60.7 ± 6.4 dB	50.9 ± 6.4 dA	10.3 ± 3.3 aC	7.3 ± 3.4 aB	6.0 ± 1.7 aA
Pinobanksin acetate/Dimethyl kaempferol isomer 1	48.2 ± 1.7 cC	43.3 ± 1.3 cA	46.1 ± 1.2 cB	5.1 ± 0.6 aC	4.3 ± 0.3 aA	4.2 ± 0.1 aB
Pinobanksin acetate/Dimethyl kaempferol isomer 2	32.6 ± 1.5 dA	33.8 ± 1.2 dA	35.2 ± 0.3 dA	2.2 ± 0.3 aA	1.8 ± 0.1 aA	1.6 ± 0.1 aA
Isosakuranetin/5-O-Methylpinobanksin	18.2 ± 1.0 bB	18.8 ± 1.0 bB	16.6 ± 1.0 bA	3.8 ± 0.6 aB	3.2 ± 0.3 aB	2.9 ± 0.2 aA
Hydroxy methoxyphenylpropenyl pinobanksin/Pinocembrin-5-O-3-hydroxy-4-methoxyphenylpropionate	18.0 ± 2.3 cA	16.8 ± 1.7 cA	15.0 ± 1.0 cA	2.3 ± 0.8 aA	1.7 ± 0.6 aA	0.9 ± 0.7 aA
Pinobanksin-3-O-pentanoate isomer 2	21.7 ± 2.2 bA	21.0 ± 2.0 bA	19.7 ± 0.7 bA	3.0 ± 1.0 aA	2.5 ± 0.7 aA	1.6 ± 0.6 aA
**Other flavonoids**						
Rhamnetin/Methylquercetin isomer 1	85.6 ± 3.6 bA	88.4 ± 3.4 bA	91.1 ± 2.0 bA	13.1 ± 2.9 aA	11.3 ± 2.1 aA	9.5 ± 1.7 aA
Apigenin	30.3 ± 2.2 bA	30.2 ± 1.7 bA	30.0 ± 0.9 bA	3.0 ± 1.0 aA	2.4 ± 0.7 aA	1.8 ± 0.4 aA
Kaempferol	28.9 ± 1.4 dB	31.4 ± 1.8 dB	27.2 ± 2.5 dA	4.5 ± 1.3 aB	3.6 ± 0.9 aB	2.8 ± 0.8 aA
Isokaempferide/Diosmetin	28.3 ± 1.4 bA	28.4 ± 1.0 bA	28.5 ± 0.4 bA	4.1 ± 1.0 aA	3.4 ± 0.6 aA	2.9 ± 0.5 aA
Rhamnetin/Methylquercetin isomer 2	15.5 ± 0.8 cB	25.0 ± 4.6 cB	15.6 ± 4.5 cA	6.6 ± 2.4 aB	5.0 ± 1.7 aB	3.4 ± 1.6 aA
Dimethylquercetin/Eupaletin	47.3 ± 3.8 bB	46.8 ± 3.0 bAB	42.0 ± 2.1 bA	7.2 ± 2.3 aB	5.6 ± 1.8 aAB	3.9 ± 1.3 aA
Pinostrobin/Medicarpin/Alpinetin isomer 1	21.0 ± 2.1 cC	18.5 ± 1.8 cB	16.5 ± 1.1 cA	2.6 ± 0.8 aC	1.9 ± 0.6 aB	1.6 ± 0.4 aA
Chrysin	17.0 ± 2.6 cA	59.5 ± 27.8 cB	15.3 ± 0.9 cA	1.5 ± 0.6 aA	1.1 ± 0.4 aB	0.7 ± 0.3 aA
Sakuranetin	15.3 ± 1.8 bA	14.5 ± 1.0 bA	13.6 ± 0.8 bA	2.4 ± 0.5 aA	2.0 ± 0.4 aA	1.6 ± 0.3 aA
Acacetin/Calycosin/Thevetiaflavone/Biochanin A/5-O-Methylgalangin/Pterosonin E/6-Methoxybaicalein isomer 1	14.0 ± 1.9 cA	12.9 ± 1.2 cA	11.3 ± 1.1 cA	1.5 ± 0.6 aA	1.1 ± 0.4 aA	0.6 ± 0.3 aA
Pinocembrin	27.8 ± 1.4 cA	28.2 ± 1.2 cAB	29.4 ± 0.9 cB	2.6 ± 0.5 aA	2.2 ± 0.3 aAB	1.8 ± 0.2 aB
Apigenin isomer	21.7 ± 2.6 cB	21.2 ± 1.8 cB	18.6 ± 1.0 cA	1.9 ± 0.8 aB	1.3 ± 0.5 aB	0.8 ± 0.4 aA
Kaempferide	16.3 ± 1.9 bB	16.0 ± 1.5 bB	14.1 ± 1.1 bA	2.7 ± 1.0 aB	2.1 ± 0.8 aB	1.4 ± 0.6 aA
Acacetin/Calycosin/Thevetiaflavone/Biochanin A/5-O-Methylgalangin/Pterosonin E/6-Methoxybaicalein isomer 2	18.3 ± 1.9 cA	17.7 ± 1.6 cA	16.7 ± 0.9 cA	1.8 ± 0.7 aA	1.4 ± 0.5 aA	1.0 ± 0.3 aA
Pinostrobin/Medicarpin/Alpinetin isomer 2	0.3 ± 0.1 aB	0.3 ± 0.1 aAB	0.2 ± 0.1 aA	0.5 ± 0.2 aB	0.3 ± 0.2 aAB	0.2 ± 0.1 aA
Vestitol/Neovestitol	13.9 ± 1.5 cB	13.2 ± 1.3 cB	11.7 ± 1.0 cA	2.0 ± 0.8 aB	1.5 ± 0.5 aB	1.0 ± 0.4 aA

Values: means ± standard deviations. Different letters represent the level of significance: *p* < 0.05. Lowercase letters indicate differences between treatments and capital letters indicate differences between digestion times.

## Data Availability

All the data generated by this research have been included in the article. For any assistance, it is possible to contact with the corresponding authors.
